# Effect of a Steaming Treatment on the Alpha-Glucosidase Inhibitory Components in the Brown Alga *Sargassum fusiforme*

**DOI:** 10.3390/molecules29246000

**Published:** 2024-12-19

**Authors:** Xinxin Liu, Yipeng Gu, Yihao Zhou, Ruiqi Zhang, Tomoyuki Koyama

**Affiliations:** 1 Graduate School of Marine Science and Technology, Tokyo University of Marine Science and Technology, 4-5-7 Konan, Tokyo 108-8477, Japangoole1020@163.com (Y.G.);; 2 Guangxi Key Laboratory of Health Care Food Science and Technology, Institute of Food Science and Technology, Hezhou University, Hezhou 542899, China

**Keywords:** brown algae, *Sargassum fusiforme*, α-glucosidase inhibitor, pyropheophytin, steaming treatment

## Abstract

The brown alga *Sargassum fusiforme* (SF) is historically consumed as a food material in Japan. A steaming process is often required for SF products on the market due to their moderate hardness and astringent taste. This investigation aimed to elucidate the effect of steaming on the anti-diabetic activity of SF and its related chemical components. Acetone extracts of SF were prepared after it were steamed for 0, 1, 2, or 4 h (SF-0h, SF-1h, SF-3h, and SF-4h, respectively). Alpha-glucosidase inhibitory profiles of each SF extract were made based on activity-guided separation. The active fractions were collected and NMR was applied for a further chemical composition analysis. Our results suggested that total polyphenol levels decreased drastically after steaming, which resulted in a drop in α-glucosidase inhibitory activity. The fatty acid, pheophytin a, and pyropheophytin a contents were elevated significantly after steaming, which contributed to the majority of the activity of steamed SF (SF-1h). However, prolonging the steaming time did not significantly affect the activity of SF further since the content of free fatty acids in steamed SF (SF-2h and SF-4h) almost did not change with a longer time of steaming. Moreover, palmitic acid, 8-octadecenoic acid, and tetradecanoic acid were identified as the top three important fatty acids for the inhibition of α-glucosidase by steamed SF. Further molecular docking results revealed that these fatty acids could interact with residues of α-glucosidase via hydrogen bonds, salt bridges, and hydrophobic interactions. In conclusion, steaming altered the α-glucosidase inhibitory properties of SF by changing the contents of polyphenols, fatty acids, and chlorophyll derivatives.

## 1. Introduction

According to the International Diabetes Federation, the global diabetes prevalence in 20–79-year-olds in 2021 was estimated to be 10.5% (536.6 million people), rising to 12.2% (783.2 million) in 2045 [1]. Suppressing the postprandial blood glucose levels is one of the effective therapeutic methods for the prevention and management of diabetes, which can be achieved by inhibiting the action of carbohydrate enzymes, including α-amylase and α-glucosidase, in the digestive tract [2]. Inhibition of these enzymes results in a delay in the digestion of dietary polysaccharides to decrease the rate of glucose absorption [3]. Clinically, α-amylase and α-glucosidase inhibitors, such as acarbose, miglitol, and voglibose, are commonly used in the treatment of diabetes, even though a series of side effects like diarrhea, flatulence, and abdominal pain were reported [4,5]. Acarbose and voglibose act locally in the gastrointestinal tract with low systemic bioavailability, which is supposed to be the major cause of their gastrointestinal side effect. As for absorbable miglitol, even if the absorbed miglitol can be quickly cleared by the kidneys, its distribution in cells of various organs and persistent inhibitory activity probably cause the side effects [5]. Thus, natural α-amylase and α-glucosidase inhibitors in food materials have attracted great attention due to their safety and feasibility. Daily intake of these components was expected to prevent and improve diabetes.

Marine seaweeds are widely recognized as an rich source of tremendous bioactive compounds due to their abundance and diversity. The anti-diabetic potential of seaweeds is acknowledged for its abundant bioactive components, such as bromophenols [6], phlorotannins, polysaccharides, fucosterol, fucoxanthin, etc. [7]. 

*Sargassum fusiforme* (SF), a perennial warm-temperature alga, belongs to the Sargassaceae family of the Fucales, Phaeophyta. It is mainly distributed along the coastline of China, Korea, and Japan [8]. Especially in Japan, it has been historically consumed as a part of the daily diet [9]. A variety of compounds from SF have been shown to exhibit anti-diabetic activity. Especially, the polysaccharide fucoidan derived from SF improved insulin resistance and the mitochondrial function of skeletal muscle in type 2 diabetic rats, and it could restore the beneficial composition of the gut flora, which was closely related to the improvement of diabetes [10]. In addition, fucoxanthin, the most abundant carotenoid in brown algae, was reported to be able to decrease the fasting blood glucose level and increase the plasma insulin level, which resulted from the regeneration of pancreatic beta cells [11]. The regulation of adipocytokine mRNA expression in white adipose tissue by a fucoxanthin supplement also played an important role in the improvement of glucose homeostasis in obese/diabetic KK-A^y^ mice [12,13]. Notably, it was found that the phlorotannins constitute up to 6% of the dry weight of SF [14]. These phlorotannins not only inhibited α-glucosidase but also induced protein kinase B and AMP-activated protein kinase [15]. The water-soluble components with potential anti-diabetic activity in SF, mainly fucoidan, have been studied well. Moreover, the activity of fucoidan is usually stable during steaming. However, a great variety of nonpolar substances with anti-diabetic activity have not been studied extensively, especially their changes during steaming.

To attenuate its astringent taste and improve its storability, SF has long been consumed after steaming or boiling rather than in its fresh form in Asia [16]. However, the effect of a steaming treatment on the anti-diabetic properties of SF has not been studied yet. Therefore, this study aimed to investigate the effect of different steaming times on the α-glucosidase and α-amylase inhibitory activities of SF. The enzyme-inhibitory activities of SF were compared after steaming for different times. Thereafter, the active fractions in each SF sample were separated and identified by NMR and GC/MS further to clarify the relationship between the chemical composition and α-glucosidase inhibitory activity. 

## 2. Materials and Methods

### 2.1. Chemicals and Reagents

Hydrogen chloride methanol reagent and *p*-nitrophenyl-α-D-glucopyranoside (*p*NPG) were purchased from Tokyo Chemical Industry Co., Ltd. (Tokyo, Japan). Analytical-grade hexane, ethyl acetate, methanol, potassium phosphate monobasic (KH_2_PO_4_), potassium phosphate dibasic (K_2_HPO_4_), sodium carbonate (Na_2_CO_3_), potassium iodide (KI), soluble starch, iodine (I_2_), methanol-d4 (MeOD), and chloroform-d1 (CDCl_3_) were purchased from Kanto Chemical Co., Inc. (Tokyo, Japan). α-Amylase from porcine pancreas and acarbose were purchased from Sigma Aldrich (St. Louis, MO, USA), while α-glucosidase was purchased from Oriental Yeast Co., Ltd. (Tokyo, Japan). Folin–Ciocalteau phenol reagent was purchased from Merck (Darmstadt, Germany). All chemicals and reagents used were of analytical grade.

### 2.2. Sample Collection and Preparation

Different batches of dried SF were provided by Takaki Shoten Corp. (Kumamoto, Japan). The materials were dried in the sun without a steaming process after harvesting on shore in Nagasaki Prefecture. The provided SF was cleaned of extraneous materials and washed thoroughly with tap water to remove salt in the laboratory. To prepare steamed SF samples, the washed SF from different batches was mixed evenly and separated randomly into 4 parts that were subjected to steaming for 0, 1, 2, or 4 h (SF-0h, SF-1h, SF-2h, and SF-4h). The broth was discarded to obtain each steamed SF sample in a colander. Thereafter, steamed SF was washed with tap water again and freeze-dried. The dried SF samples were ground into powders and stored for subsequent use.

### 2.3. Preparation of the Crude Extract

The three extracts of fresh SF (no steaming) were prepared individually as described below. The hexane extract was prepared by soaking 5 g of SF sample in 100 mL of hexane. The mixture was allowed to stand at room temperature for 24 h and it should be blocked from light during extraction. Then, the total filtrate was concentrated to dryness in a vacuum to obtain the hexane extract. The same extraction procedure was applied with acetone and methanol to obtain acetone and methanol extracts, respectively. The yields were calculated as the ratio of the dried extract weight to the dried sample weight. Each crude extract was applied to thin layer chromatography (TLC) (ODS RP-18WF_254_S, Art.1.13124, Merck KGaA, Germany) and was tested in the α-glucosidase and α-amylase inhibition assays.

### 2.4. α-Glucosidase Inhibition Assay

The assay was performed as described previously [17], with minor modifications. In short, fifty microliters of potassium phosphate buffer (100 mM, pH 7.0) and 20 μL of the crude extracts or fractions were mixed before the addition of 20 μL of yeast α-glucosidase (0.1 U/mL) (As). The sample was replaced with 20% DMSO and was used as the control (Ac). The enzyme was replaced with potassium phosphate buffer and was used as a blank for the samples (Bs) or a blank for the control (Bc). The mixture was pre-incubated at 37 °C for 5 min. The enzyme inhibition assay was initiated by adding 20 μL of *p*-NPG solution (1 mM). It was terminated by the addition of 50 μL of sodium carbonate (200 mM) after the mixture was incubated at 37 °C for 10 min. Enzyme activity was determined by measuring the absorbance at 405 nm with a microplate reader (Thermomax; MolecularDevices, Sunnyvale, CA, USA). The α-glucosidase inhibitory activity was calculated using the following formula: Inhibitory rate (%) = 100 × [(Ac − Bc) − (As − Bs)]/(Ac − Bc). Acarbose was used as positive control for comparison.

### 2.5. α-Amylase Inhibition Assay

The assay was conducted as reported previously [18], with small modifications. Thirty microliters of potassium phosphate buffer (100 mM, pH 6.9) and 20 μL of crude extracts or fractions were mixed before the addition of 20 μL of α-amylase (0.5 U/mL) (As). Similarly, the sample was replaced with 20% DMSO and was used as the control (Ac). The enzyme was replaced with buffer and was used as a blank for the samples (Bs) or a blank for the control (Bc). The mixture was pre-incubated at 37 °C for 5 min. The assay was initiated by adding 100 μL of the starch solution (0.5 mg/mL). It was terminated by the addition of 150 μL of the iodine solution (5 mM, containing 1 mM hydrogen chloride) after the mixture was incubated at 37 °C for 10 min. Enzyme activity was determined by measuring the absorbance at 590 nm with a microplate reader (Thermomax; MolecularDevices, Sunnyvale, CA, USA). The α-glucosidase inhibitory activity was calculated using the following formula: Inhibitory rate (%) = 100 × [(Bc − Ac) − (Bs − As)]/(Bc − Ac). Acarbose was used as positive control for comparison.

### 2.6. Total Polyphenol Content

Folin–Ciocalteau phenol reagent (Merck KGaA) was used for the measurement of the total polyphenol content in the acetone extract of SF, as described previously [19]. Fifty microliters of extract (dissolved in 20% methanol) at 125 μg mL^−1^ was mixed with 50 μL of Folin–Ciocalteau phenol reagent. After standing at room temperature for 10 min, 100 μL of a 15% Na_2_CO_3_ solution was added. Thereafter, the mixture was incubated in the dark for 30 min and the absorbance at 650 nm was measured. Gallic acid was used as the standard, and the total phenolic content was expressed as micrograms of gallic acid equivalents (GAE) per milligram of sample.

### 2.7. Fucoxanthin and Chlorophyll Derivative Contents

The identification and quantification of the fucoxanthin and chlorophyll derivatives in the acetone extracts were performed using a Waters e2695 high-performance liquid chromatography (HPLC) system coupled with a Waters 2998 photodiode array detector (Waters Corporation, Milford, MA, USA). The column used for chromatographic separation was a NOMURA Develosil ODS-HG-5 column (250 mm × 4.6 mm, 5 μm) (Kanagawa, Japan). Twenty microliters of the acetone extract of SF (2 mg/mL) was injected for the HPLC analysis. The mobile phase consisted of two kinds of eluents: a mixture of methanol and distilled water (90:10, *v*:*v*) as eluent A, and a mixture of methanol and ethyl acetate (75:25, *v*:*v*). The flow rate was set at 1 mL/min for the entire run with the following program: 0–20 min (eluent A) and 20–50 min (eluent B). The identification of fucoxanthin (Fucox, 8.53 min), chlorophyll c (Chl c, 14.96 min), chlorophyll a (Chl a, 28.40 min), chlorophyll a′ (Chl a′, 28.88 min), pheophytin a (Pheo a, 34.13 min), pheophytin a′ (Pheo a′, 35.19 min), and pyropheophytin a (Pyro a, 41.57 min) was performed after comparing their retention time sand UV-vis spectra with those of the standards. The fucoxanthin standard was purchased from FUJIFILM Wako Pure Chemical Co. (Osaka, Japan). The Chl c, Chl a, Pheo a, and Pyro a standards used for quantification were purified from fraction 10 (SF-0h), fraction 45 (SF-0h), fraction 66 (SF-1h), and fraction 82 (SF-4h), respectively. The quantification of each component was based on the calibration curve of Fucox (450 nm), Chl c (435 nm), Chl a (435 nm), Pheo a (410 nm), and Pyro a (410 nm). The results were expressed in micrograms (μg) per milligram of acetone extract (μg/mg extract).

### 2.8. Free Fatty Acid Composition Analysis

Free fatty acid methyl esterification was performed before the GC-MS analysis for a better chromatographic resolution, as described previously [20] with minor modifications. Briefly, 50 microliters of the acetone extract of SF (10 mg/mL) was added with 10 μL of 1% pentanoic acid (internal standard) and then mixed with 1 mL of hydrogen chloride methanol reagent (containing 5~10% hydrogen chloride). After heating at 55 °C for 15 min, the methylated fatty acids were eluted by adding 1 mL of hexane to the mixture. The hexane layer was taken and a 3 μL aliquot was subjected to a GCMS-QP2010Plus (Shimadzu, Kyoto, Japan) in splitless mode, coupled with an Rtx-5MS capillary column (30 m × 0.25 mm, 0.25 µm, Restek, Bellefonte, PA, USA). Helium was used as the carrier gas at a constant flow rate of 1.5 mL/min. The column temperature was maintained at 40 °C for 2 min, then increased to 140 °C at a rate of 10 °C/min, followed by increasing to 250 °C at a rate of 5 °C/min, and held at 250 °C for 3 min. The injector and the detector temperatures were set at 240 °C and 200 °C, respectively, and the solvent cut time was set at 6 min. The identification of peaks was based on matching the mass spectra with those in the library for the National Institute of Standards and Technology (NlST3208 and NIST 08s). The content of each fatty acid was expressed as the relative folds of pentanoic acid in SF-0h, SF-1h, SF-2h, and SF-4h, and the total fatty acid content of SF-0h was normalized to 1.

### 2.9. α-Glucosidase Inhibitory Profiling of SF

Given that the crude acetone extract showed the most potent inhibition of α-glucosidase, crude extracts of each steamed SF were prepared with acetone using the same procedure, and they were further fractionated using column chromatography. In brief, the acetone extract of each SF (70 mg) was subjected to a YAMAZEN ODS-SM column (50 μm, 3.0 × 16.5 cm) equipped with a pump 540 (YAMAZEN Corp., Osaka, Japan). The column was run by a mobile phase with gradient elution at a flow rate of 8 mL/min. Eluent A (methanol/distilled water = 90:10) was used from the beginning to 25 min, eluent B (methanol/ethyl acetate = 90:10) was used from 25 min to 55 min, and finally, eluent C (ethyl acetate) was used from 55 min to the end. Fractions were collected at 8 mL/min/vial to give 90 fractions. Twenty microliters of each fraction was dried in a vacuum and was redissolved in 200 μL of 20% dimethyl sulfoxide (DMSO) for further α-glucosidase inhibition assays.

### 2.10. Analysis of the Components with Inhibitory Activity in the Fractions

To estimate the chemical compositions of the active fractions, Frac. 4, Frac. 66, and Frac. 82 collected were dissolved in MeOD or CDCl_3_ for the measurement of ^1^H (600 MHz) and ^13^C (150 MHz) with a Bruker AVANCE III HD 600 MHz spectrometer (Bruker Biospin, Rheinstetten, Germany). The spectra obtained were compared with those reported in the literature. To identify the potential α-glucosidase inhibitors in the bioactive Frac. 43, Frac. 43 (7.2 mg) collected from the separation of SF-4h was subjected to a YAMAZEN ODS-SM column (50 μm, 3.0 × 16.5 cm) for further separation. It was first eluted with a mixture of methanol and distilled water (*v*:*v* = 95:5) at a rate of 8 mL/min from the beginning to 80 min, and then eluted with ethyl acetate from 80 min to 95 min. Four subfractions were collected: Frac. 43-1 (0–30 min, 0.3 mg), Frac. 43-2 (30–60 min, 1.2 mg), Frac. 43-3 (60–80 min, 1.6 mg), and Frac. 43-4 (80–95 min, 4.5 mg). Each subfraction was applied for further NMR measurements.

### 2.11. Random Forest Analysis of Fatty Acids

To better clarify the importance of various free fatty acids in the fractions of SF with the α-glucosidase inhibitory activity, free fatty acids were identified and quantified from Frac. 37 to Frac. 57. Eleven kinds of fatty acids identified by GC-MS were selected for evaluation, and the α-glucosidase inhibitory activities of Frac. 37 to Frac. 57 were selected as response values. To estimate the importance of certain fatty acids for the α-glucosidase inhibitory activity of the acetone extract of SF, percentage increases in the mean squared error (MSE%) of variables were used: higher MSE% values imply more important variables [21].

### 2.12. Molecular Docking

Molecular docking between the top three fatty acids (palmitic acid, 8-octadecenoic acid, and tetradecanoic acid) and α-glucosidase was performed as described previously [22] to study their interactions. The structure of α-glucosidase (PDB ID: 3A4A) was obtained from the RCSB PDB website, and the three-dimensional structures of palmitic acid, 8-octadecenoic acid, and tetradecanoic acid were downloaded from the PubChem website (PubChem), https://pubchem.ncbi.nlm.nih.gov/, accessed on 15 November 2024. Non-H-bonding hydrogen atoms and Gasteiger charges were added by AutoDock tools (version: 1.5.7, Scripps Research, San Diego, CA, USA), and molecular docking was processed by AutoDock Vina (Version 1.2.2, Scripps Research, USA). The energies of molecular docking, the positions of the hydrogen bonds, and the hydrophobic interactions between α-glucosidase and the inhibitor were analyzed. The interaction between the inhibitor and α-glucosidase from the AutoDock output files was analyzed by Protein-Ligand Interaction Profiler (PLIP) [23]. PyMOL Molecular Graphics System (Version 2.5.5, Schrödinger, Inc., New York, NY, USA) was used to visualize the results of molecular docking.

### 2.13. Statistical Analysis

All experiments were performed at least three times with three different batches of samples. The data are expressed as means ± standard deviations (SDs). Statistical analyses comparing between groups were determined using one-way analysis of variance. The differences were further evaluated using Tukey’s multiple comparison tests and considered significant at *p* < 0.05. All statistical analyses were performed using SPSS 22.0 (SPSS Inc., Chicago, IL, USA).

## 3. Results

### 3.1. Effect of the Extraction Solvent on Carbohydrate-Digesting Enzyme Inhibition by SF

**Figure 1:**
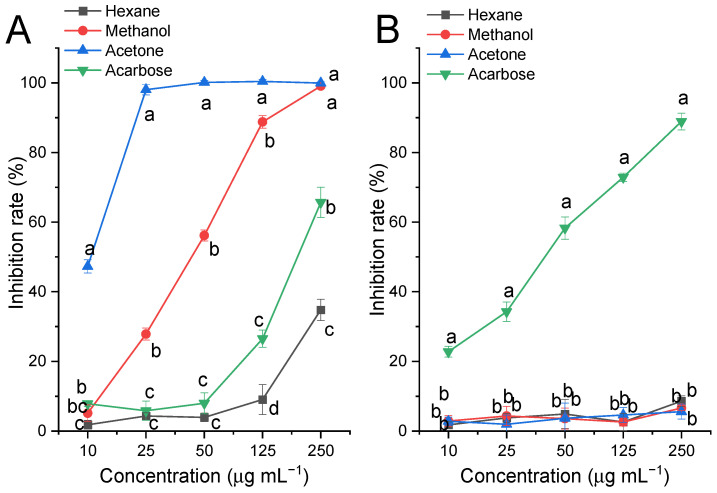
Effect of the extraction solvent on carbohydrate-digesting enzyme inhibition by SF-0h. (**A**) α-Glucosidase, each extract and acarbose were prepared at 10, 25, 50, 125, and 250 μg mL^−1^ for the assay; (**B**) α-amylase, each extract and acarbose were prepared at 10, 25, 50, 125 and 250 μg mL^−1^ for the assay. Values are presented as means (SDs), *n* = 3. ^abcd^ *p* < 0.05, different letters indicate significant differences among different extracts at the same concentration.

Hexane, methanol, and acetone extracts of fresh SF (no steaming), and acarbose were prepared and subjected to α-glucosidase and α-amylase inhibition assays. As shown in Figure 1A, each extract of SF exhibited potent inhibitory activity against α-glucosidase, especially the acetone extract; it inhibited almost 100% of α-glucosidase activity even at 50 μg mL^−1^, which was significantly more potent than acarbose. However, all extracts showed very limited inhibitory activity against α-amylase at a concentration of 1000 μg mL^−1^ when compared to acarbose, as shown in Figure 1B. Thus, the further experiments were focused mainly on the α-glucosidase inhibitory activity of the acetone extract of SF.

### 3.2. Effect of the Extraction Solvent on the Phytochemical Compositions and Free Fatty Acids in SF

**Figure 2:**
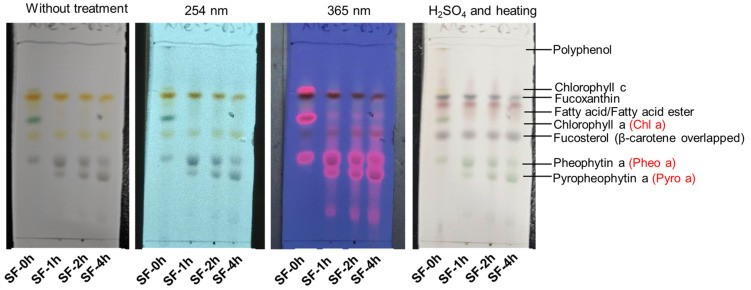
Thin layer chromograph pattern of the acetone extracts of SF (SF-0h, -1h, -2h, and -4h). An ODS plate was developed with methanol and ethyl acetic (85:15, *v*:*v*). The developed spots were detected with UV (254 or 365 nm) and with 10% sulfuric acid followed by heating at 220 °C.

As shown in Table 1, the total polyphenol content of steamed SF (SF-1h, SF-2h, and SF-4h) decreased significantly when compared with fresh SF (SF-0h). Fucoxanthin levels also decreased time-dependently. Additionally, most of the chlorophylls (Chl c, Chl a, and Chl a′) decomposed after 1 h of steaming treatment. On the other hand, Pheo a and Pheo a′ increased firstly after 1 h of steaming, while they decreased gradually with the extension of the steaming time. Pyro a could not be detected in SF-0h, while it increased markedly during steaming. The changes in these phytochemical components could be estimated by thin-layer chromatography, as shown as well in Figure 2. The spots of Chl c and Chl a almost disappeared in SF-1h, SF-2h, and SF-4h. The spot of Pheo a became thicker (SF-1h) first and then became thinner (SF-2h and SF-4h) with the extension of steaming. The trend of Pyro a was also consistent with the results of phytochemical composition analysis by HPLC.

**Figure 3:**
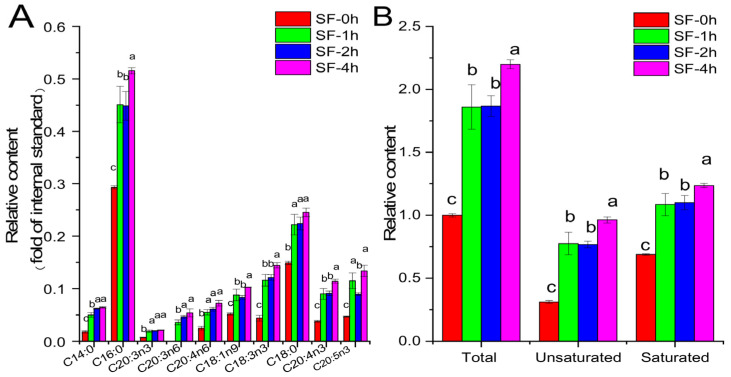
Free fatty acid analysis of the acetone extract of SF. (**A**) Free fatty acid composition; (**B**) relative contents of total, unsaturated, and saturated free fatty acids. The free fatty acid content was expressed as the fold of the internal standard pentanoic acid. The total fatty acid content of SF-0h was normalized to 1. Values are presented as means (SDs), *n* = 3. ^abc^ *p* < 0.05, different letters indicate significant differences.

Free fatty acids in the acetone extracts of SF were quantified, as shown in Figure 3. All detected free fatty acids in SF increased significantly after steaming treatment. Especially, tetradecanoic acid levels increased by 186.40%, 251.80%, and 265.72%, respectively, after 1 h, 2 h, and 4 h of steaming treatment. The major free fatty acid in the acetone extract of SF was palmitic acid, and its levels also increased by 54.07%, 53.11%, and 75.96%, respectively, after 1 h, 2 h, and 4 h of steaming treatment. Generally, steaming treatment increased the total free fatty acid content in the acetone extract of SF. Among them, the unsaturated free fatty acid and saturated free fatty acid contents of SF-4h increased by 201.06% and 79.35%, respectively, when compared with those of SF-0h.

### 3.3. Effect of Steaming on α-Glucosidase Inhibition and the Yield of SF

**Figure 4:**
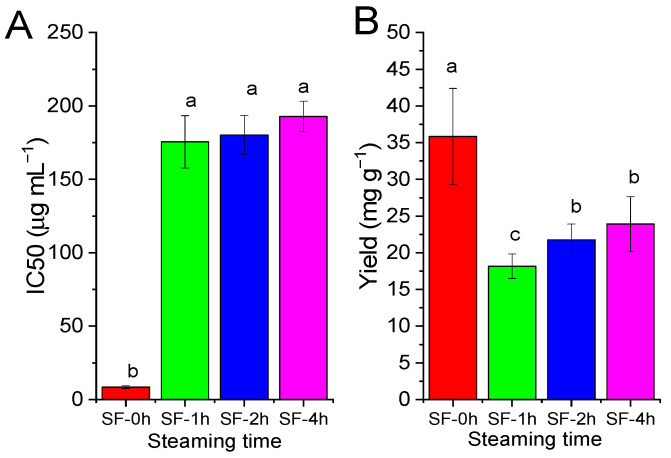
Effect of steaming on alpha-glucosidase inhibition and the yield of SF. (**A**) IC_50_ of the acetone extract of SF against α-glucosidase. Fresh SF was steamed for 0, 1, 2, or 4 h and extracted with acetone. (**B**) Yield of the acetone extract (mg) of SF (g). Values are presented as means (SDs), *n* = 3. ^abc^ *p <* 0.05, different letters indicate significant differences.

As shown in Figure 4, one hour of steaming treatment significantly decreased the α-glucosidase inhibitory activity of fresh SF (SF-1h). However, prolonging the steaming time to 4 h did not affect the IC_50_ of SF significantly (*p* > 0.05). One hour of steaming decreased the yield of SF significantly (*p* < 0.05), as the yield of SF-1h decreased by 49.31%. Even if there was no statistically significant difference, prolonging the steaming time increased the yield slightly (SF-2h and SF-4h) when compared with that of SF-1h.

### 3.4. Effect of Steaming on the α-Glucosidase Inhibitory Profile of SF

**Figure 5:**
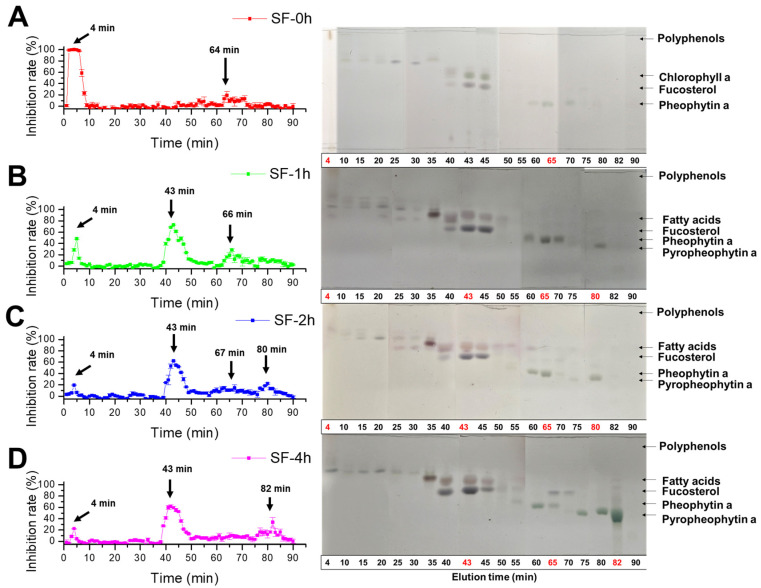
Effect of the steaming time on the α-glucosidase inhibitory profile of SF. (**A**) SF-0h; (**B**) SF-1h; (**C**) SF-2h; (**D**) SF-4h. SF was steamed for 0, 1, 2, or 4 h, and extracted with acetone; each acetone extract of SF was separated on the ODS column. The fractions were collected and applied to the α-glucosidase inhibition assay and developed by TLC on an ODS plate with methanol and ethyl acetic (*v*:*v* = 85:15). The spots were detected with 10% sulfuric acid and heated at 220 °C subsequently.

To identify which component contributed to the majority of the α-glucosidase inhibitory activity of SF, acetone extracts of SF were prepared after various times of steaming treatment and subjected to an ODS column for separation while monitoring the inhibitory activity. As shown in Figure 5A, fractions eluted at around 4 min were suggested to contain high levels of polar component(s) on TLC with the highest inhibitory activity. The active component(s) were estimated to be phlorotannin derivatives based on the NMR data of the fraction (Figure S1). However, the activities of the fractions at 4 min decreased greatly with the extension of the steaming time, as shown in Figure 5B–D. In steamed SF extracts (SF-1h, SF-2h, and SF-4h), the most of activity was contributed by the fraction eluted at 43 min, which was composed of two main components based on the pattern shown in a thin layer chromograph (Figure 5B). Additionally, a peak appeared at 66 min that was identified as Pheo a (Figure S2) and increased first when the steaming time was 1 h (SF-1h), whereas it decreased gradually when the steaming time was extended to 4 h (SF-4h). On the contrary, a new peak appeared at around 82 min, which was identified as Pyro a based on the ^1^H-NMR spectrum (Figure S3).

### 3.5. Identification of the Active Components of Frac. 43

**Figure 6:**
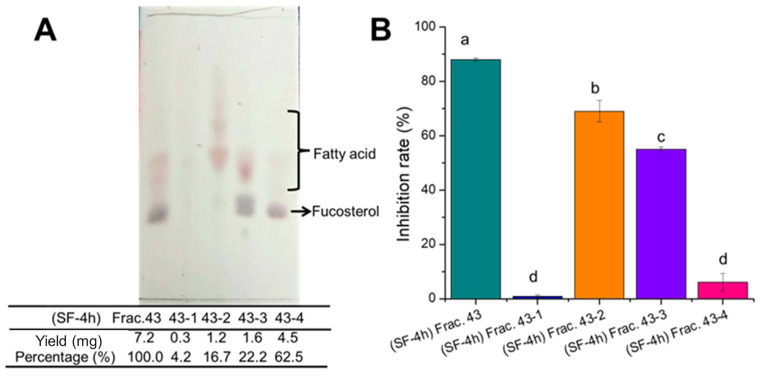
Identification of the active components of Frac. 43. (**A**) TLC results for Frac. 43 and the subfractions. (**B**) α-Glucosidase inhibition by Frac. 43 and its subfractions. Acetone extracts of SF steamed for 0, 1, 2, or 4 h were subjected to the ODS column for separation, and Frac. 43 (appeared at 43 min) of SF-4h was further separated into 4 subfractions (Frac. 43-1, 43-2, 43-3, and 43-4); each fraction was dried and redissolved in an equal volume of 20% DMSO for the subsequent α-glucosidase inhibition assay. Values are presented as means (SDs), *n* = 3. ^abcd^ *p* < 0.05, different letters indicate significant differences.

To identify which component possesses the α-glucosidase inhibitory activity in Frac. 43 (appeared at 43 min), Frac. 43 of SF-4h was further separated into 4 subfractions (Figure 6A) and redissolved in an equal volume of 20% DMSO after drying for the α-glucosidase inhibition assay. According to the ^1^H NMR data, it was estimated that Frac. 43-2 and Frac. 43-3 contain several types of fatty acids, and Frac. 43-3 and Frac. 43-4 contain sterol-related compound(s) as major components (Figure S4). And the Frac. 43-4 shown as a single spot on the TLC plate was identified as fucosterol based on the NMR data (Figure S5). As shown in Figure 6B, fucosterol (Frac. 43-4) presented extremely weak activity against alpha-glucosidase, whereas the Frac. 43-2 and Frac. 43-3 showed potent inhibitory activities. Therefore, fatty acids were suggested to be active components in Frac. 43. 

### 3.6. Free Fatty Acid Determination and Variable Importance Evaluation

**Figure 7:**
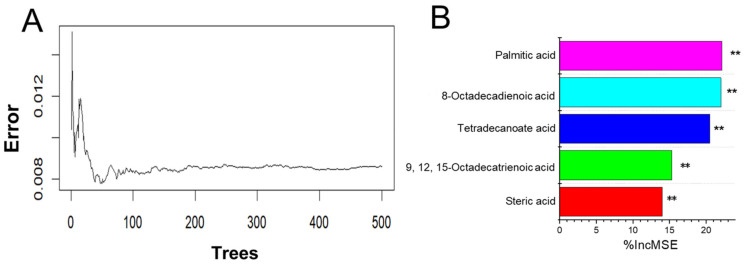
Variable importance of free fatty acids by the random forest model. (**A**) Cumulative error rates of the random forest classification. (**B**) Top 5 significant features identified by random forest. The accuracy importance measure was computed for each tree and averaged over the forest (150 trees). Percentage increases in the mean squared error (MSE%) of variables were used to estimate the importance of predictors, and the higher the MSE% was, the more important the predictors were. ** *p* < 0.01.

Given that the active components in Frac. 43 were a series of free fatty acids, the detailed free fatty acid compositions of Frac. 37 to Frac. 57 were measured after methylation and were analyzed by a random forest model to better understand the effects of different free fatty acids on the alpha-glucosidase inhibitory activity of SF. All the free fatty acids determined were selected for the random forest model. The classification tree for this free fatty acid classification was set to 500. As shown in Figure 7, the cumulative classification error steadily dropped as the number of trees rose and finally stabilized at 0.008. The top three significant variables in the random forest classification model were palmitic acid (C16:0), 8-octadecenoic acid (C18:1n9), and tetradecanoic acid (C14:0).

### 3.7. Molecular Docking of Alpha-Glucosidase and Fatty Acids

**Figure 8:**
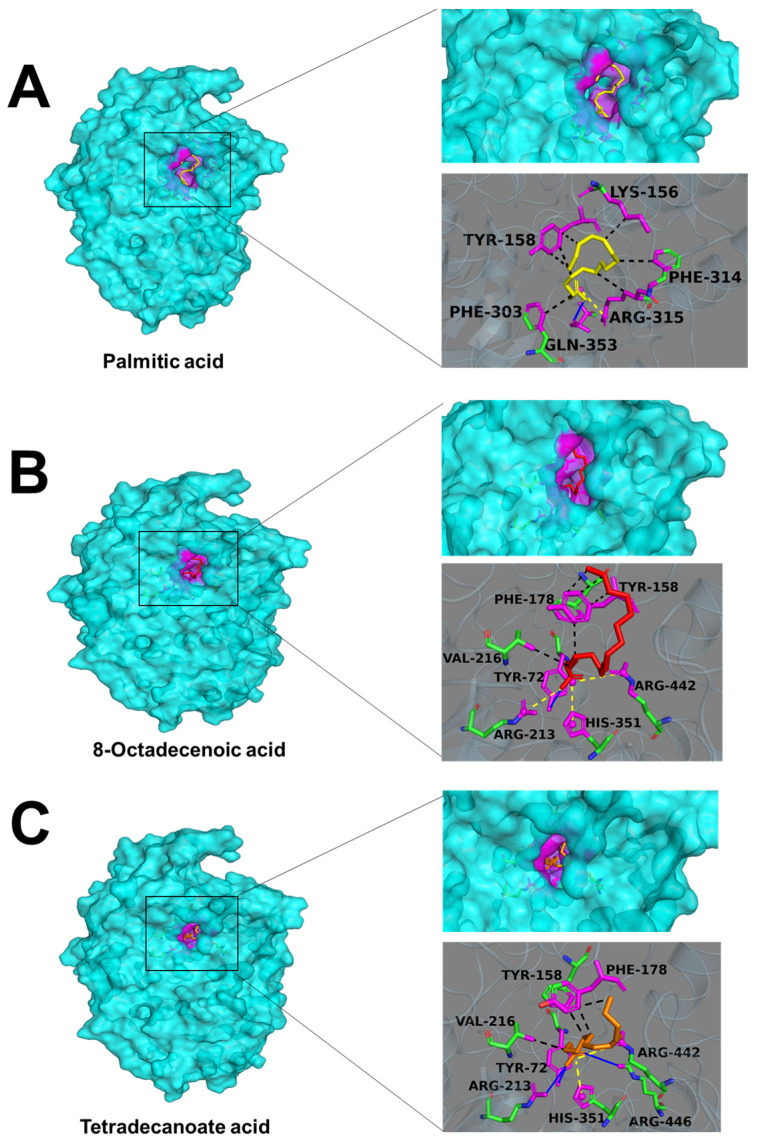
Conformational and interaction analyses of molecular docking for alpha-glucosidase and fatty acids. (**A**) Palmitic acid; (**B**) 8-octadecenoic acid; (**C**) tetradecanoic acid. The expansion of the highlighted regions shows a close-up view of the binding sites and binding residues. The blue solid line represents a hydrogen bond, the yellow dotted line represents a salt bridge, and the black dotted line represents a hydrophobic interaction.

The top three free fatty acids (palmitic acid, 8-octadecenoic acid, and tetradecanoic acid) analyzed by random forest model were applied to molecular docking to predict their modes of binding. The most stable conformation in the binding of small molecules to α-glucosidase with the lowest binding energies was presented in Figure 8. Palmitic acid, 8-octadecenoic acid, and tetradecanoic acid exhibited great affinity for alpha-glucosidase with binding energies of 5.2, 6.2, and 5.0 kcal/mol, respectively. All these three kinds of fatty acids were buried totally in the active cavity. Palmitic acid interacted with the Lys156, Tyr158, Phe303, and Phe314 of α-glucosidase through hydrophobic interactions. Additionally, palmitic acid interacted through one hydrogen bond at Arg315 and one salt bridge at Gln353 of α-glucosidase. Similar hydrophobic interactions with α-glucosidase could be found in 8-octadecenoic acid and tetradecanoic acid as well. However, compared with palmitic acid, 8-octadecenoic acid formed more salt bridges with the α-glucosidase (Arg213, His351, and Arg442), and tetradecanoic acid formed three hydrogen bonds (Tyr72, Arg213, and Arg442) and two salt bridges (His351 and Arg442).

## 4. Discussion

Growing evidence has linked carbohydrate-digesting enzyme inhibitors in food materials with the improvement of diabetic conditions. Therefore, the potential of marine seaweed and the contained ingredients for antidiabetic medication have attracted great attention [24,25,26]. *Sargassum fusiforme* has been consumed as a sea vegetable in Japan for centuries. Modern research reveals that SF presents great potential in the treatment of diabetes, and it exerts its antidiabetic action through a variety of approaches [27,28]. Compared to other commercial edible algae, steaming or boiling treatment is necessary for SF to attenuate its astringent taste and improve its storability, whereas the effect of these thermal treatments on anti-diabetic properties and related chemical components of SF has not been studied yet. Therefore, we investigated the effect of the steaming time on the α-glucosidase inhibitory activity of SF.

Based on the present results, steaming treatment decreased the α-glucosidase inhibitory activity of SF to a large extent, which could be proven by the decrease in the α-glucosidase inhibitory activity of the crude acetone extract (Figure 2) and the change in the α-glucosidase inhibitory profile (Figure 3). These results indicated that the loss of α-glucosidase inhibitory activity after steaming was highly related to the reduction in the polyphenols in SF. In previous studies, it has been widely assumed that different cooking methods (boiling, steaming, frying, etc.) will induce changes in the total polyphenol content in foods [29,30]. The effects of cooking methods on total polyphenol contents varies greatly among different food materials since they depend on two opposite phenomena. Regarding seaweed, a significant loss of the polyphenol content for the alga *Undaria pinnatifida* (Wakame) and a slight increase for the alga *Laminaria* sp. (Kombu) were observed after they were boiled for 20 min and 1 h, respectively [31]. Moreover, it has been reported that the total polyphenol content of the alga *Himanthalia elongate* decreased by 32.06% after steaming for 45 min, which could be due to the leaching of nutrients in water, since a reduction in the amount of extract obtained could be observed as well [32]. Similar results were observed in this study, as the total polyphenol content of SF decreased time-dependently and the yield decreased significantly.

In addition to polyphenols, marine algae-derived natural pigments, including chlorophyll a, pheophytin a, β-carotene, and fucoxanthin, have been reported to provide multiple health benefits [33]. Fucoxanthin, one of the most extensively studied algae-specific pigments, has been widely reported for its outstanding inhibitory properties against α-amylase and α-glucosidase [26,34,35]. Unfortunately, our results suggested that it made a relatively limited contribution to the α-glucosidase inhibitory activity of SF based on the current conditions (Figure 5A). Thermal treatment induces the conversion of Chl a to Pheo a due to the release of magnesium, and prolonging the heating time induces the further conversion of pheophytin a to pyropheophytin a [36]. We also observed that a great amount of Pheo a formed after SF was steamed for 1 h. It has been reported that the porphyrin rings of both Chl a and Pheo a could interact with amino acid residues of α-amylase and α-glucosidase to change the characteristics of enzymes, thereby inhibiting their activities [22]. Thus, the formation of Pheo a induced a slight increase in α-glucosidase inhibition at 66 min in the α-glucosidase inhibitory profile of SF-1h. Pheo a converted to Pyro a when more rigorous thermal treatment was applied. The peak inhibition that appeared at 82 min in the α-glucosidase inhibitory profile of SF-4h was supposed to be owing to the formation of Pyro a.

Marine algae are rich in polyunsaturated fatty acids (PUFAs) and are of potential value as sources of essential fatty acids [37]. Marine algae-derived fatty acids exert anti-diabetic actions through various approaches. For instance, it has been pointed out that omega-3 fatty acids, especially marine n-3 PUFAs (eicosapentaenoic acid (C20:5n-3, EPA) and docosahexaenoic acid (C22:6n-3, DHA)), could improve insulin sensitivity in humans [38]. Furthermore, the PUFA-rich extracts of the microalgae *Chlorella pyrenoidosa* and *Spirulina platensis* altered the composition of the gut microbiota and elevated the diversity of the gut bacterial community of diabetic rats [39]. Moreover, a variety of fatty acids have been demonstrated to be able to inhibit α-glucosidase activity [40,41,42]. Three saturated and unsaturated fatty acids, including cerotic acid, n-octacos-9-enoic acid, and 11-eicosenoic acid, isolated from the brown alga *Dictyopteris hoytii* showed inhibitory activity against α-glucosidase [43]. Chun-Han Su et al. compared the inhibitory properties of 10 fatty acids against α-glucosidase and α-amylase [40]. Their results revealed that all tested fatty acids showed weak α-amylase inhibition, whereas the majority of them presented potent α-glucosidase inhibition. Moreover, the unsaturated fatty acids presented more potent inhibitory activity against α-glucosidase than that of saturated fatty acids. Therefore, the presence of a double bond may play a crucial role in the inhibition of α-glucosidase. Other researchers also reported similar results, and they suggested that fatty acids with an odd number of double bonds showed stronger α-glucosidase inhibition than those with an even number of double bonds [44]. Here, we observed that the contents of almost all fatty acids increased significantly in the acetone extract (Figure 3). The increase in the free fatty acid content was mainly due to the leaching of water-soluble substances to water during heating. As a result, the percentage of fatty acids in the SF extract increased. Moreover, the de-esterification of esterified lipid during heating probably contributed to the increase in the free fatty acid content in the SF extract as well. Compared to saturated fatty acids, the increased content of unsaturated fatty acids was more notable. The top three fatty acids (palmitic acid, 8-octadecenoic acid, and tetradecanoic acid) that played the most important roles in the inhibition of α-glucosidase were evaluated by a random forest analysis. Palmitic acid is the most dominant fatty acid in microalgae [20]. Its inhibition of α-glucosidase has been extensively studied by several in vitro and in silica research methods [44,45]. Asp214, Glu276, and Asp349 are the catalytic triad of the active site in the α-glucosidase molecule, and targeting these could considerably affect the enzyme activity [45]. Our molecular docking results revealed that palmitic acid, 8-octadecenoic acid, and tetradecanoic acid entered the catalytic site of α-glucosidase deeply and showed great affinity. Even though these three fatty acids did not interact with Asp214, Glu276, and Asp349 directly in our molecular docking analysis, their close interactions with nearby residues of catalytic triad through a hydrogen bond, salt bridge, and hydrophobic interactions were supposed to affect the activity of α-glucosidase.

## 5. Conclusions

In this study, an acetone extract of SF was prepared and applied in a α-glucosidase inhibition assay after SF was steamed for 0, 1, 2, or 4 h. SF-0h presented potent inhibitory activity against α-glucosidase and this property was altered a lot after the steaming treatment. In particular, polyphenols in SF-0h presented extremely potent inhibitory activity against α-glucosidase, which contributed to the most inhibitory activity against α-glucosidase. However, steaming induced the loss of total polyphenols in SF significantly. Abundant free fatty acids in steamed SF (SF-1h, SF-2h, and SF-4h) contributed to the major activity, which was the most notable change in the α-glucosidase inhibitory activity of SF during steaming. Additionally, steaming resulted in the conversion of Chl a to Pheo a and Pyro a, which also affected the α-glucosidase inhibitory profile of SF to some extent, especially palmitic acid, 8-octadecenoic acid, and tetradecanoic acid, because they were identified as the top three important fatty acids for the inhibition of α-glucosidase. However, prolonging the steaming time did not significantly affect the inhibitory activity nor the profiles of SF (SF-2h and SF-4h). Furthermore, the molecular docking results revealed that these fatty acids could enter the catalytic sites of α-glucosidase and probably had close interactions with the nearby residues via hydrogen bonds, salt bridges, and hydrophobic interactions. In a word, one hour of steaming treatment decreased the α-glucosidase inhibitory activity of SF greatly due to the loss of polyphenols. Prolonging the steaming time would not significantly affect the α-glucosidase inhibitory activity of SF.

## Figures and Tables

**Table 1:** Phytochemical compositions of the acetone extracts of SF.

	SF-0h	SF-1h	SF-2h	SF-4h
Total polyphenols ^$^	419.96 ± 13.05 ^a^	131.42 ± 5.14 ^b^	110.97 ± 4.30 ^c^	114.79 ± 1.72 ^c^
Fucoxanthin	9.60 ± 0.74 ^b^	12.17 ± 0.57 ^a^	8.39 ± 1.10 ^b^	3.75 ± 0.73 ^c^
Chlorophyll c	11.86 ± 0.30	-	-	-
Chlorophyll a	55.30 ± 4.56 ^a^	3.70 ± 2.05 ^b^	-	-
Chlorophyll a′	2.80 ± 0.60	-	-	-
Pheophytin a	24.15 ± 2.85 ^d^	132.62 ± 4.48 ^a^	101.88 ± 1.39 ^b^	58.16 ± 8.23 ^c^
Pheophytin a′	7.63 ± 0.77 ^d^	37.30 ± 1.78 ^a^	27.43 ± 2.55 ^b^	18.00 ± 1.78 ^c^
Pyropheophytin a	-	45.25 ± 1.98 ^c^	81.64 ± 2.44 ^b^	122.08 ± 17.84 ^a^

^$^: Total polyphenols are expressed as micrograms of gallic acid equivalents (GAE) per milligram dry mass of the acetone extract. Other components are expressed as micrograms per milligram dry mass of the acetone extract. ^abcd^* p* < 0.05, different letters indicate significant differences among different extracts.

## Data Availability

Data available on request from the authors.

## Supplementary Materials

The following supporting information can be downloaded at https://www.mdpi.com/article/10.3390/molecules29246000/s1, Table S1. Relative free fatty acid content in acetone extract of SF; Figure S1. Analysis of the component(s) in Frac. 4 by HMQC and HMBC experiments; Figure S2. Identification of a compound in Frac. 66 with the 1H-NMR spectrum; Figure S3. Identification of a compound in Frac. 82 with the 1H-NMR spectrum; Figure S4. Analysis of components in Fracs. 43-2, 43-3, and 43-4 with 1H-NMR spectra; Figure S5. Identification of a compound in Fraction 43-4 by NMR experiments.

## References

[1] Sun H., Saeedi P., Karuranga S., Pinkepank M., Ogurtsova K., Duncan B.B., Stein C., Basit A., Chan J.C., Mbanya J.C. (2022). IDF Diabetes Atlas: Global, regional and country-level diabetes prevalence estimates for 2021 and projections for 2045. Diabetes Res. Clin. Pract..

[2] Santos C.M., Proença C., Freitas M., Araújo A.N., Silva A.M., Fernandes E. (2022). Inhibition of the carbohydrate-hydrolyzing enzymes α-amylase and α-glucosidase by hydroxylated xanthones. Food Funct..

[3] Li X., Bai Y., Jin Z., Svensson B. (2022). Food-derived non-phenolic α-amylase and α-glucosidase inhibitors for controlling starch digestion rate and guiding diabetes-friendly recipes. LWT.

[4] Martin A.E., Montgomery P.A. (1996). Acarbose: An α-glucosidase inhibitor. Am. J. Health-Syst. Pharm..

[5] Reuser A.J.J., Wisselaar H.A. (1994). An evaluation of the potential side-effects of α-glucosidase inhibitors used for the management of diabetes mellitus. Eur. J. Clin. Investig..

[6] Jacobtorweihen J., Spiegler V. (2023). Phylogenetic distribution of bromophenols in marine algae and the generation of a comprehensive bromophenol database. Phytochem. Rev..

[7] Hakim M.M., Patel I.C. (2020). A review on phytoconstituents of marine brown algae. Future J. Pharm. Sci..

[8] Zhang R., Zhang X., Tang Y., Mao J. (2020). Composition, isolation, purification and biological activities of Sargassum fusiforme polysaccharides: A review. Carbohydr. Polym..

[9] Liu J., Luthuli S., Yang Y., Cheng Y., Zhang Y., Wu M., Choi J.i., Tong H. (2020). Therapeutic and nutraceutical potentials of a brown seaweed Sargassum fusiforme. Food Sci. Nutr..

[10] Jia R.-B., Li Z.-R., Wu J., Ou Z.-R., Liao B., Sun B., Lin L., Zhao M. (2020). Mitigation mechanisms of Hizikia fusifarme polysaccharide consumption on type 2 diabetes in rats. Int. J. Biol. Macromol..

[11] Oliyaei N., Moosavi-Nasab M., Tamaddon A.M., Tanideh N. (2021). Antidiabetic effect of fucoxanthin extracted from Sargassum angustifolium on streptozotocin-nicotinamide-induced type 2 diabetic mice. Food Sci. Nutr..

[12] Maeda H., Hosokawa M., Sashima T., Miyashita K. (2007). Dietary combination of fucoxanthin and fish oil attenuates the weight gain of white adipose tissue and decreases blood glucose in obese/diabetic KK-Ay mice. J. Agric. Food Chem..

[13] Hosokawa M., Miyashita T., Nishikawa S., Emi S., Tsukui T., Beppu F., Okada T., Miyashita K. (2010). Fucoxanthin regulates adipocytokine mRNA expression in white adipose tissue of diabetic/obese KK-Ay mice. Arch. Biochem. Biophys..

[14] Li Y., Fu X., Duan D., Liu X., Xu J., Gao X. (2017). Extraction and identification of phlorotannins from the brown alga, Sargassum fusiforme (Harvey) Setchell. Mar. Drugs.

[15] Kang S.-y., Kim E., Kang I., Lee M., Lee Y. (2018). Anti-diabetic effects and anti-inflammatory effects of Laminaria japonica and Hizikia fusiforme in skeletal muscle: In vitro and in vivo model. Nutrients.

[16] Hwang E.K., Park C.S., Hwang E.K., Park C.S. (2020). Seaweed cultivation and utilization of Korea. Algae.

[17] Shang C., Gu Y., Koyama T. (2021). Major triterpenes, cycloeucalenone and 31-norcyclolaudenone as inhibitors against both α-glucosidase and α-amylase in banana peel. Int. J. Food Sci. Technol..

[18] Xiao Z., Storms R., Tsang A. (2006). A quantitative starch? Iodine method for measuring alpha-amylase and glucoamylase activities. Anal. Biochem..

[19] Salahuddin M.A.H., Othman Z., Ying J.C.L., Noor E.S.M., Idris S. (2017). Antioxidant activity and phytochemical content of fresh and freeze-dried Lepisanthes fruticosa fruits at different maturity stages. J. Agric. Sci.

[20] Sahu A., Pancha I., Jain D., Paliwal C., Ghosh T., Patidar S., Bhattacharya S., Mishra S. (2013). Fatty acids as biomarkers of microalgae. Phytochemistry.

[21] Breiman L. (2001). Random forests. Mach. Learn..

[22] Wang X., Yang Z., Shen S., Ji X., Chen F., Liao X., Zhang H., Zhang Y. (2023). Inhibitory effects of chlorophylls and its derivative on starch digestion in vitro. Food Chem..

[23] Adasme M.F., Linnemann K.L., Bolz S.N., Kaiser F., Salentin S., Haupt V.J., Schroeder M. (2021). PLIP 2021: Expanding the scope of the protein–ligand interaction profiler to DNA and RNA. Nucleic Acids Res..

[24] Zaharudin N., Staerk D., Dragsted L.O. (2019). Inhibition of α-glucosidase activity by selected edible seaweeds and fucoxanthin. Food Chem..

[25] Kim K.-T., Rioux L.-E., Turgeon S.L. (2014). Alpha-amylase and alpha-glucosidase inhibition is differentially modulated by fucoidan obtained from Fucus vesiculosus and Ascophyllum nodosum. Phytochemistry.

[26] Lordan S., Smyth T.J., Soler-Vila A., Stanton C., Ross R.P. (2013). The α-amylase and α-glucosidase inhibitory effects of Irish seaweed extracts. Food Chem..

[27] Zheng Q., Jia R.-B., Ou Z.-R., Li Z.-R., Zhao M., Luo D., Lin L. (2022). Comparative study on the structural characterization and α-glucosidase inhibitory activity of polysaccharide fractions extracted from Sargassum fusiforme at different pH conditions. Int. J. Biol. Macromol..

[28] Wang X., Huang C., Fu X., Jeon Y.-J., Mao X., Wang L. (2023). Bioactivities of the Popular Edible Brown Seaweed Sargassum fusiforme: A Review. J. Agric. Food Chem..

[29] Palermo M., Pellegrini N., Fogliano V. (2014). The effect of cooking on the phytochemical content of vegetables. J. Sci. Food Agric..

[30] Gunathilake K.P.P., Ranaweera K.S., Rupasinghe H.V. (2018). Effect of different cooking methods on polyphenols, carotenoids and antioxidant activities of selected edible leaves. Antioxidants.

[31] Amorim K., Lage-Yusty M.-A., López-Hernández J. (2012). Changes in bioactive compounds content and antioxidant activity of seaweed after cooking processing. CyTA-J. Food.

[32] Cox S., Abu-Ghannam N., Gupta S. (2012). Effect of processing conditions on phytochemical constituents of edible Irish seaweed Himanthalia elongata. J. Food Process. Preserv..

[33] Pangestuti R., Kim S.-K. (2011). Biological activities and health benefit effects of natural pigments derived from marine algae. J. Funct. Foods.

[34] Kawee-Ai A., Kim A.T., Kim S.M. (2019). Inhibitory activities of microalgal fucoxanthin against α-amylase, α-glucosidase, and glucose oxidase in 3T3-L1 cells linked to type 2 diabetes. J. Oceanol. Limnol..

[35] Yin S., Siahaan E.A., Niu L., Shibata M., Liu Y., Hagiwara T. (2023). Real time monitoring and evaluation of the inhibition effect of fucoxanthin against α-amylase activity by using QCM-A. Front. Nutr..

[36] Östbring K., Rayner M., Sjöholm I., Otterström J., Albertsson P.-Å., Emek S.C., Erlanson-Albertsson C. (2014). The effect of heat treatment of thylakoids on their ability to inhibit in vitro lipase/co-lipase activity. Food Funct..

[37] Lu Q., Li H., Xiao Y., Liu H. (2021). A state-of-the-art review on the synthetic mechanisms, production technologies, and practical application of polyunsaturated fatty acids from microalgae. Algal Res..

[38] Zheng J.-S., Huang T., Yang J., Fu Y.-Q., Li D. (2012). Marine N-3 polyunsaturated fatty acids are inversely associated with risk of type 2 diabetes in Asians: A systematic review and meta-analysis. PLoS ONE.

[39] Wan X.-z., Li T.-t., Zhong R.-t., Chen H.-b., Xia X., Gao L.-y., Gao X.-x., Liu B., Zhang H.-y., Zhao C. (2019). Anti-diabetic activity of PUFAs-rich extracts of Chlorella pyrenoidosa and Spirulina platensis in rats. Food Chem. Toxicol..

[40] Moheimanian N., Mirkhani H., Edraki N., Poustforoosh A., Momeni S., Khalighian N., Zidorn C., Sohrabipour J., Jassbi A.R. (2024). Bioassay-guided purification of α-glucosidase inhibitor fatty acids from Padina tetrastromatica. J. Appl. Phycol..

[41] Su C.H., Hsu C.H., Ng L.T. (2013). Inhibitory potential of fatty acids on key enzymes related to type 2 diabetes. Biofactors.

[42] Seong S.H., Nguyen D.H., Wagle A., Woo M.H., Jung H.A., Choi J.S. (2019). Experimental and computational study to reveal the potential of non-polar constituents from Hizikia fusiformis as dual protein tyrosine phosphatase 1B and α-glucosidase inhibitors. Mar. Drugs.

[43] Ur Rehman N., Rafiq K., Khan A., Ahsan Halim S., Ali L., Al-Saady N., Hilal Al-Balushi A., Al-Busaidi H.K., Al-Harrasi A. (2019). α-Glucosidase inhibition and molecular docking studies of natural brominated metabolites from marine macro brown alga Dictyopteris hoytii. Mar. Drugs.

[44] Miyazawa M., Yagi N., Taguchi K. (2005). Inhibitory compounds of α-glucosidase activity from *Arctium lappa* L. J. Oleo Sci..

[45] Xie X., Chen C., Fu X. (2021). Screening α-glucosidase inhibitors from four edible brown seaweed extracts by ultra-filtration and molecular docking. LWT.

