# Supplementary material for: Effect of a Steaming Treatment on the Alpha-Glucosidase Inhibitory Components in the Brown Alga Sargassum fusiforme
Source: Molecules. 2024 Dec 19;29(24):6000. doi: 10.3390/molecules29246000 (PMC11678123; doi:10.3390/molecules29246000)
Supplement: Supplementary file 1 [file molecules-29-06000-s001.zip › molecules-3346985-supplementary.pdf]

Supplementary Materials

Effect of a Steaming Treatment on the Alpha-Glucosidase Inhibitory Components in the Brown Alga *Sargassum fusiforme*

Xinxin Liu [1], Yipeng Gu [1,2], Yihao Zhou [1], Ruiqi Zhang [1] and Tomoyuki Koyama [1,*]

[1] Graduate School of Marine Science and Technology, Tokyo University of Marine Science and Technology, 4-5-7 Konan, Tokyo 108-8477, Japan

[2] Guangxi Key Laboratory of Health Care Food Science and Technology, Institute of Food Science and Technology, Hezhou University, Hezhou 542899, China

* Correspondence: tskoyama@kaiyodai.ac.jp

Table S1. Relative free fatty acid content in acetone extract of SF

Free fatty acids	SF-0h	SF-1h	SF-2h	SF-4h
C14:0	0.017±0.002	0.050±0.004	0.062±0.001	0.064±0.002
C16:0	0.292±0.001	0.451±0.035	0.448±0.027	0.515±0.006
C20:3n3	0.007±0.003	0.018±0.005	0.019±0.001	0.021±0.001
C20:3n6	ND	0.035±0.002	0.046±0.003	0.054±0.007
C20:4n6	0.025±0.002	0.054±0.005	0.060±0.004	0.072±0.005
C18:1n9	0.047±0.003	0.088±0.005	0.083±0.004	0.102±0.001
C18:3n3	0.043±0.002	0.116±0.011	0.121±0.005	0.144±0.005
C18:0	0.149±0.006	0.222±0.011	0.224±0.012	0.245±0.008
C20:4n3	0.038±0.003	0.089±0.020	0.090±0.004	0.114±0.004
C20:5n3	0.046±0.002	0.114±0.010	0.089±0.003	0.134±0.011
Total FF	0.668±0.008	1.264±0.118	1.247±0.055	1.469±0.024
Unsaturated FF	0.208±0.007	0.517±0.058	0.512±0.017	0.643±0.016
Saturated FF	0.460±0.004	0.724±0.059	0.735±0.038	0.825±0.011

Free fatty acids content was expressed as the folds of internal standard pentanoic acid. Ten microliters of pentanoic acid (10 mg mL^{-1}) were added before methylation. Values were presented as mean (SD), n=3.

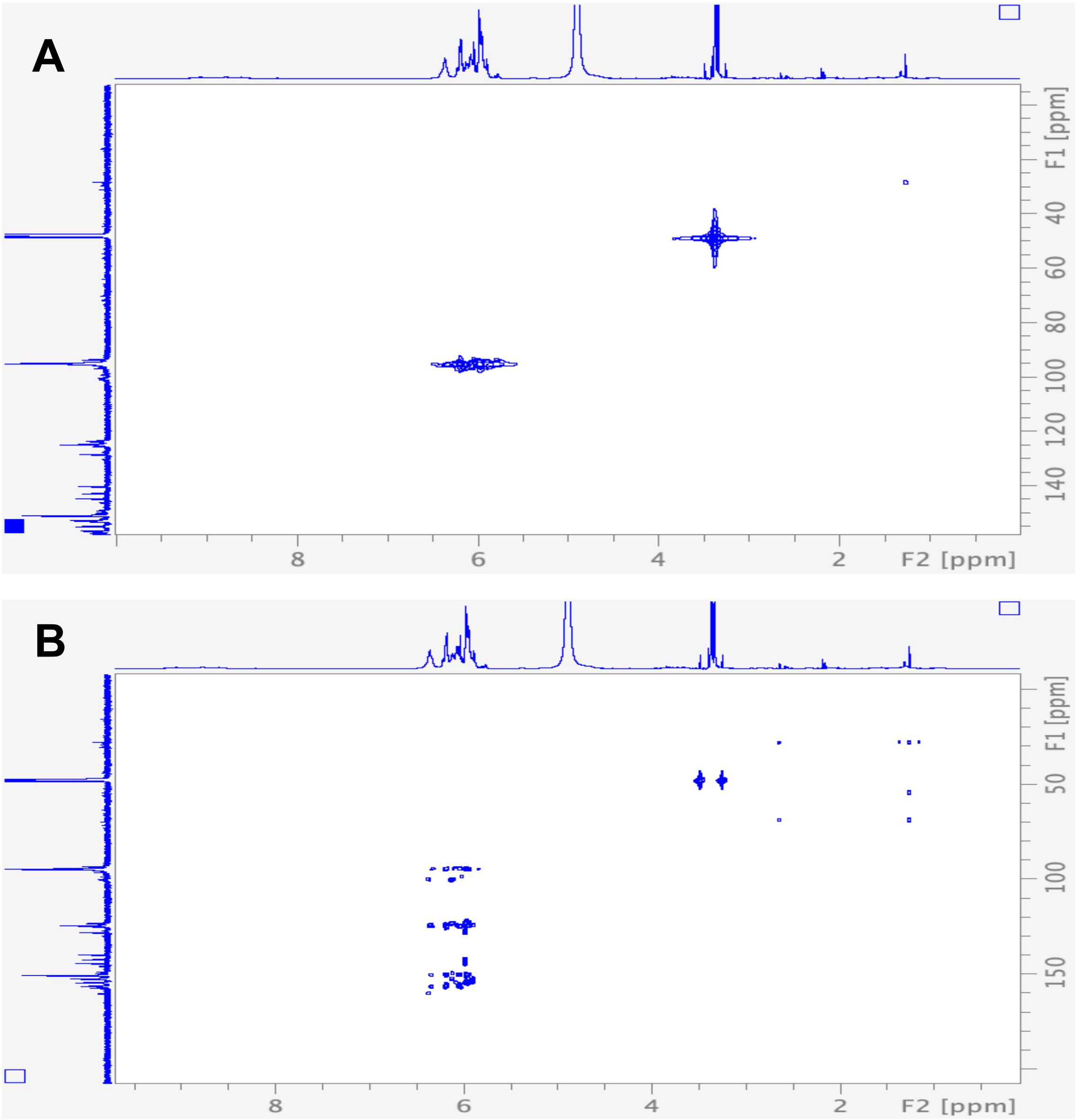

Figure S1. Analysis of component(s) in Frac. 4 by HMQC and HMBC experiments.

Based on ^{1}H and ^{13}C NMR patterns in the projections of HMQC spectrum (MeOD, 600 MHz) (A), the major component in the fraction were estimated to be aromatic compound(s). The HMQC correlations between several aromatic protons at δ 5.8-6.4 and carbons at δ 94-95 suggest the existent of these aromatic methines located between oxygenated aromatic carbons. And the HMBC correlations (MeOD, 600 MHz) (B) between these protons and aromatic carbons at δ 94-103 (C=C-H/C), 122-129 (C=C-C), 142-160 (C=C-O) indicate phloroglucinol derivative units. On the other hand, no crosspeaks between these aromatic moieties and other signals were observed. Based on these data, the major component of fraction 4 are suggested to be phlorotannin(s), which is polymer(s) of phloroglucinol.

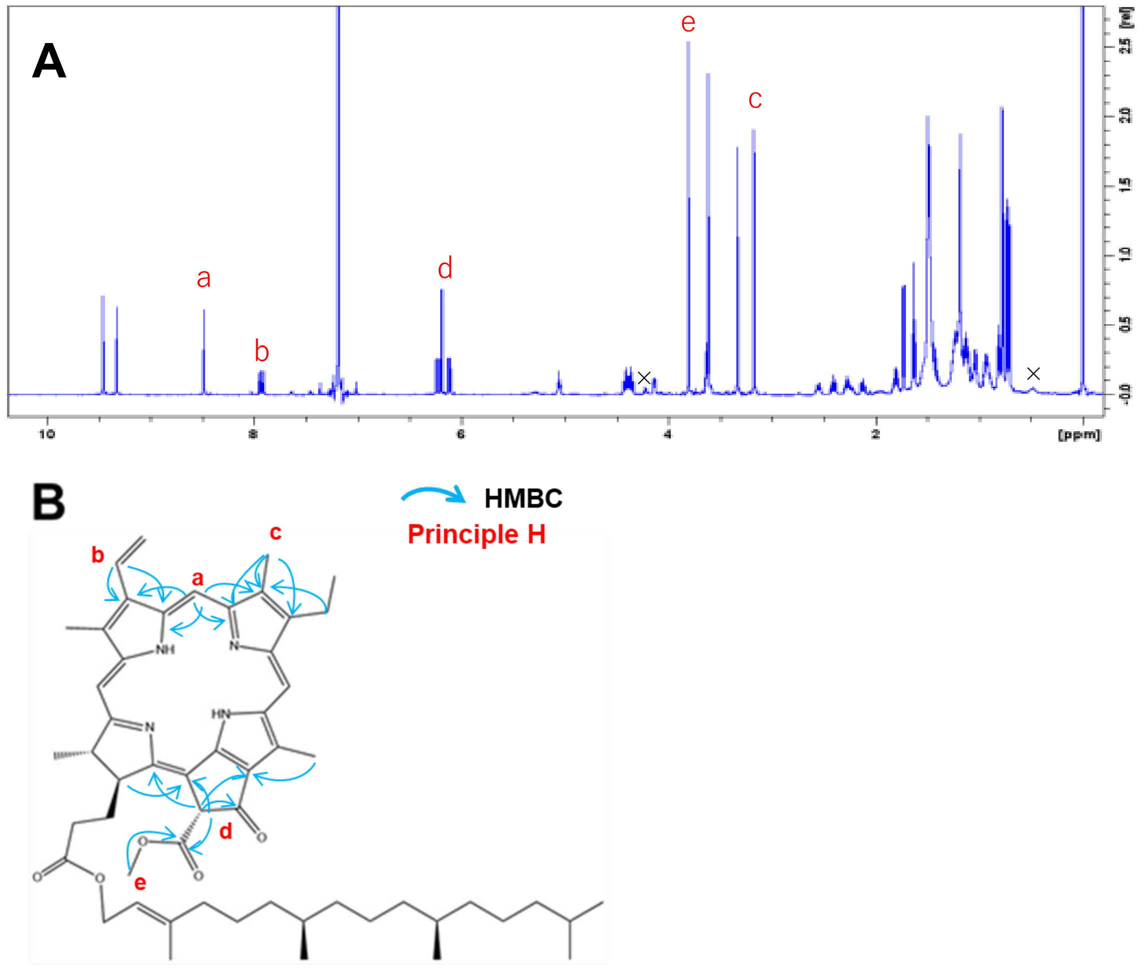

Figure S2. Identification of a compound in Frac. 66 with ^{1}H-NMR spectrum.

Based on analysis of ^{1}H-NMR data ($CDCl_3$, 600 MHz). The ^{1}H NMR ($CDCl_3$): δ 8.484 (a, s, 1H), 7.931 (b, dd, 1H, *J*= 11.5, 17.1 Hz), 6.188 (d, s, 1H), 3.806 (e, s, 3H), 3.174 (c, s, 3H). Principle H was assigned based on HMBC.

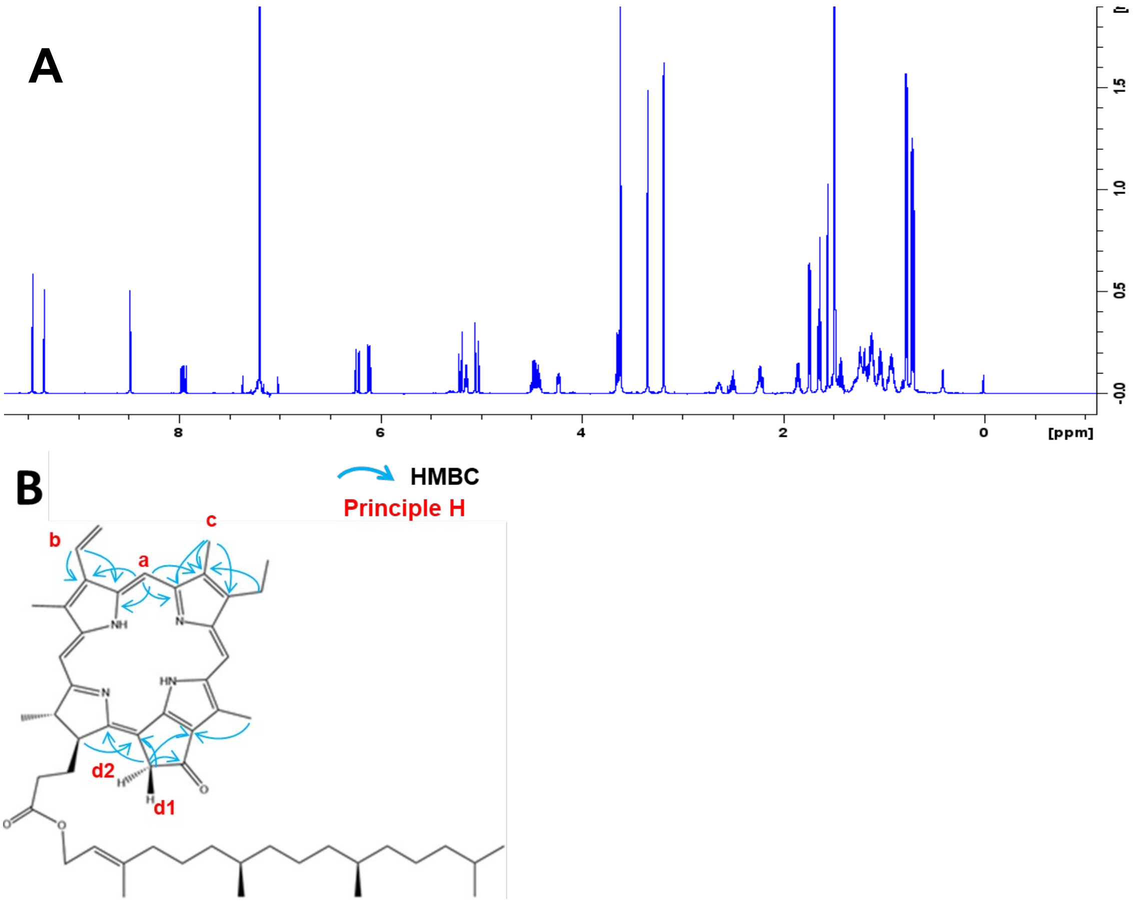

Figure S3. Identification of a compound in Frac. 82 with ^{1}H-NMR spectrum.

Based on analysis of ^{1}H-NMR data ($CDCl_3$, 600 MHz). The ^{1}H NMR ($CDCl_3$): δ 8.481 (a, s, 1H), 7.950 (b, dd, 1H, *J*= 11.5, 17.1 Hz), 5.197 (d1, d, *J*=19.1 Hz, 1H), 5.038 (d2, d, *J*=19.1 Hz, 1H), 3.18 (c, s, 3H). Principle H was assigned based on HMBC.

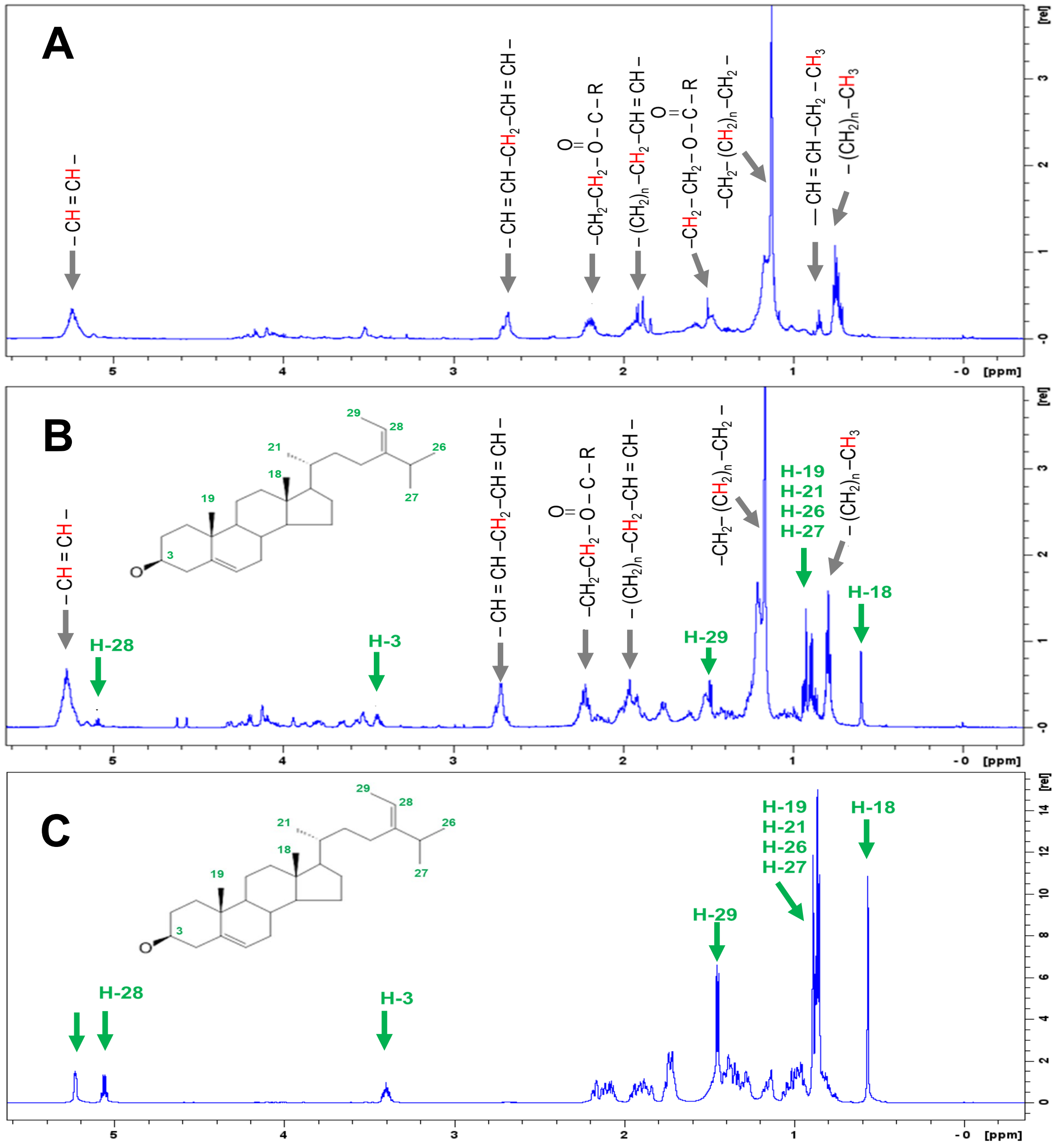

Figure S4. Analysis of components in Fracs. 43-2, 43-3, and 43-4 with ^{1}H-NMR spectra.

These ^{1}H-NMR spectra ($CDCl_3$, 600 MHz) indicated unsaturated fatty acids (gray arrows) and cholesterol derivatives (green arrows) as the major components in these three fractions. Several signals were observed commonly in these fractions. In the Frac. 43-2 (A), no cholesterol derivative was observed. In the Frac 43-3 (B) contained both major components. And 43-4 (C) contained cholesterol derivative without fatty acids.

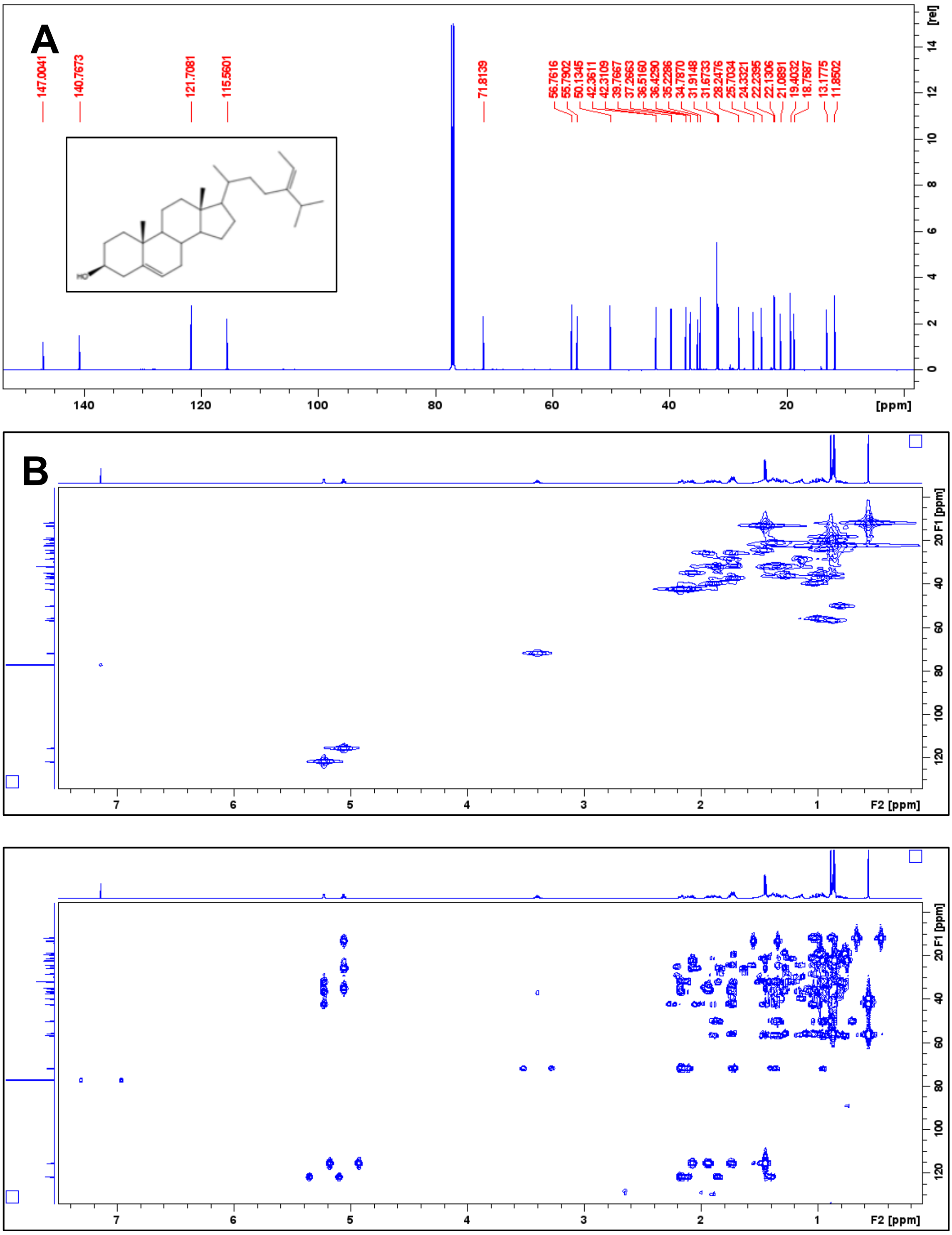

Figure S5. Identification of a compound in Fraction 43-4 by NMR experiments.

The ^{13}C-NMR spectrum ($CDCl_3$, 150 MHz) (A) of fraction 43-4 given 29 carbon signals, which were well corresponded to data of fucosterol, a typical algal cholesterol. The HMQC (B) and HMBC (C) correlations were supported the results of the identification ($CDCl_3$, 600 MHz).
